# An adiponectin receptor agonist promote osteogenesis via regulating bone‐fat balance

**DOI:** 10.1111/cpr.13035

**Published:** 2021-05-03

**Authors:** Hanghang Liu, Shibo Liu, Huanzhong Ji, Qiucheng Zhao, Yao Liu, Pei Hu, En Luo

**Affiliations:** ^1^ State Key Laboratory of Oral Disease & National Clinical Research Center for Oral Diseases & Department of Oral Maxillofacial Surgery West China Hospital of Stomatology Sichuan University Chengdu Sichuan P. R. China; ^2^ Maine Medical Center Research Institute Maine Medical Center Scarborough ME USA

**Keywords:** adiponectin receptor, bone regeneration, bone‐fat balance, stem cells

## Abstract

**Objectives:**

Adiponectin signalling has been considered to be a promising target to treat diabetes‐related osteoporosis. However, contradictory results regarding bone formation were observed due to the various isoforms of adiponectin. Therefore, it would be necessary to investigate the effect of adiponectin receptor signals in regulating bone‐fat balance.

**Materials and Methods:**

We primarily applied a newly found specific activator for adiponectin receptor, AdipoRon, to treat bone metabolism‐related cells to investigate the role of Adiponectin receptor signals on bone‐fat balance. We then established femur defect mouse model and treated them with AdipoRon to see whether adiponectin receptor activation could promote bone regeneration.

**Results:**

We found that AdipoRon could slightly inhibit the proliferation of pre‐osteoblast and pre‐osteoclast, but AdipoRon showed no effect on the viability of mesenchymal stromal cells. AdipoRon could remarkably promote cell migration of mesenchymal stromal cells. Additionally, AdipoRon promoted osteogenesis in both pre‐osteoblasts and mesenchymal cells. Besides, AdipoRon significantly inhibited osteoclastogenesis via its direct impact on pre‐osteoclast and its indirect inhibition of RANKL in osteoblast. Moreover, mesenchymal stromal stems cells showed obviously decreased adipogenesis when treated with AdipoRon. Consistently, AdipoRon‐treated mice showed faster bone regeneration and repressed adipogenesis.

**Conclusions:**

Our study demonstrated a pro‐osteogenic, anti‐adipogenic and anti‐osteoclastogenic effect of adiponectin receptor activation in young mice, which suggested adiponectin receptor signalling was involved in bone regeneration and bone‐fat balance regulation.

## Graphical abstract

Adiponectin receptors 1 and 2 are expressed early in mesenchymal stem cells lineage and in precursors including pre‐osteoblast, pre‐osteoclasts and chondrocytes. Using an adiponectin receptor agonist AdipoRon, our study demonstrated a pro‐osteogenic, anti‐adipogenic and anti‐osteoclastogenic effect of adiponectin receptor activation in young mice, which suggested adiponectin receptor signalling was involved in bone regeneration and bone‐fat balance regulation.

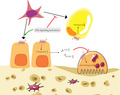

## 1 INTRODUCTION

Adiponectin (APN) is a major adipokine secreted by adipocytes, and it signals via activating adiponectin receptors 1 and 2 (AR1 and AR2). AR1 and AR2 were majorly expressed in skeletal muscle and liver, respectively.^1^ Besides, AR1 and AR2 were also reported to be expressed early in mesenchymal stem cells (MSC) lineage and in precursors including pre‐osteoblast, pre‐osteoclasts and chondrocytes, suggesting that ARs signalling may be also involved in bone and cartilage metabolism.^2^

Although ARs signals are considered to be a promising target to prevent or treat osteoporosis and several studies investigated the role of APN on bone regeneration and bone‐fat balance, contradictory results were reported. Most of the in vitro studies showed that the activation of ARs by APN could promote the osteogenesis of both osteoblast and osteoblast progenitors^3^, ^4^, ^5^, ^6^; meanwhile, APN was reported to inhibit osteoclastogenesis directly via AR1 on osteoclast precursors^7^ or indirectly via downregulating RANKL secreted from osteoblasts.^8^ APN also showed therapeutic efficacy in oestrogen deficiency‐induced osteoporosis and diabetes‐induced implant destabilization.^9^, ^10^ However, few studies showed the opposite results that APN may stimulate cellular plasticity of osteoblasts towards adipocytes^11^; APN may also induce RANKL and repress the expression of OPG in osteoblasts via activating AR1‐p38 pathway.^12^ Several pre‐clinical and clinical studies indicated higher serum adiponectin levels were negatively correlated with bone mineral density,^13^, ^14^ adiponectin transgenic mice showed lower femur bone mineral content.^15^ Reasons for the discrepancy remain unclear; several hypotheses were reported including the complex endocrine feedback regulation of APN and indirect effects of APN via mediating sympathetic tone, insulin sensitivity and energy homeostasis.

Therefore, the application of a specific activator of ARs would be more promising to study the effect of ARs signalling in bone regeneration and bone‐fat balance in a cell‐autonomous manner. AdipoRon (APR) is a newly identified adiponectin receptor agonist which could specifically bind and activate AR1 and AR2 and exert similar antidiabetic effects of APN through AMPK and PPAR‐α pathway.^16^ APR was found to improve glucose intolerance as well as insulin resistance in high‐fat diet mice model.^16^ Few studies also suggested that APR could increase the survival, migration of bone marrow mesenchymal stem cells (BMSCs),^17^ promote diabetic fracture repair and alveolar bone regeneration.^18^, ^19^ However, it is still lacking sufficient investigations regarding the effect of APR on the regulation of bone‐fat balance.

In this study, we conducted series of experiments to investigate the effect of APR on bone‐fat balance including osteoblast‐osteoclast and osteoblast‐adipocyte differentiation balance to clarify the potential role of ARs activation during bone regeneration, and subsequently try to provide more clues and have a comprehensive understanding for the biological effect of adiponectin and ARs signalling.

## 2 MATERIALS AND METHODS

### 2.1 Reagents

AdipoRon was purchased from Selleck Chemicals, China. MC3T3‐E1 and RAW264.7 cell lines were provided by State Key Laboratory of Oral Disease, West China Hospital of Stomatology, Sichuan University.

### 2.2 Cell culture and AR1, AR2 detection

4‐week‐old Sprague Dawley (SD) Rat was used for the isolation of BMSCs, tibiae and femurs were collected and cut in the ends, then spun by centrifuge, complete α‐MEM (low glucose α‐MEM that consisted of 1% Penicillin‐Streptomycin and 10% foetal bovine serum (FBS)) was used to resuspend the cells. Adipocyte‐derived stem cells (ADSCs) were isolated from 4‐week‐old Sprague Dawley (SD) Rat, inguinal adipose tissues were collected and cut into small pieces, and then digested with type I collagenase for 1 hour at 37℃ water bath. The digested cells were spun by centrifuge at 500 g for 5 min and then re‐suspended in complete α‐MEM. The third passage of BMSCs and ADSCs was used for our future experiment. ADSCs identification was performed by flow cytometry via detecting surface marker including CD, 31, CD29, CD45 and CD44. BMSCs identification was performed via detecting surface marker including CD29, CD24, CD45 and CD90. MC3T3‐E1 cell line was cultured with complete α‐MEM, and RAW264.7 was cultured with complete DMEM (high glucose DMEM that consisted of 1% Penicillin‐Streptomycin and 10% FBS). Cells were passaged when they reached 80%‐90% confluence.

For the detection of AR1 and AR2, cells were seeded on 12‐well plates for immunofluorescence staining. 4% paraformaldehyde was used for cell fixation, and 0.5% Triton X‐100 was used for permeabilization. Goat serum was used for non‐specific antigens blocking. Samples were then incubated with related primary antibodies overnight at 4℃. FITC‐ and Cy3‐conjuncted secondary antibody were used to react with AR1 and AR2 primary antibody, respectively.

### 2.3 Osteogenesis, osteoclastogenesis and adipogenesis induction

For osteogenic differentiation, MC3T3‐E1, BMSCs and ADSCs were routinely cultured with as we described before.^2^ Briefly, induction medial that consisted of complete αMEM, 50μg/mL ascorbic acid, 100 nM dexamethasone and 10 mM beta‐glycerophosphate was used for osteogenic differentiation. Medium was changed every two days. Cells were treated with or without APR combined with osteogenic medium for 3‐7 days to perform alkaline phosphatase (ALP) staining and ALP quantitative (Alkaline Phosphatase Assay Kit, Beyotime, China), or for 14 days to conduct Alizarin red staining. AR‐stained cells were destained with 10% cetylpyridinium chloride at room temperature for 1 hour, and the absorbance was measured at 570 nm for quantitative.

For osteoclastogenesis, RAW264.7 and subsequent osteoclastogenesis differentiation was culture as we previously described.^20^ Complete high glucose DMEM contained 50 ng/mL murine recombinant sRANK Ligand (Proteintech, US) was used for the induction of osteoclastogenesis. Tartrate‐resistant acid phosphatase (TRAP) staining and TRAP quantitative was performed on day 6 with or without APR treatment using TRAP/ALP stain kit (Wako, Japan) and Tartrate‐Resistant Acid Phosphatase Assay Kit (Beyotime, China) according to the manufactures’ instructions. The size and number of TRAP + cells were measured by Image J; five random fields were used for cell counting.

For adipogenic differentiation, BMSCs and ADSCs were cultured as we described before.^2^ Adipogenic differentiation included two stages: induction stage and maintain stage. In induction stage, complete high glucose DMEM containing 1 µM Rosiglitazone, 500 nM IBMX, 1 µM Dexamethasone and 10 µg/mL insulin was used for 3 days. Base medium that consisted of complete high glucose DMEM, 10 µg/mL insulin and 1 µM Rosiglitazone was used for maintaining stage for 6 days. Oil Red O (ORO) staining and related quantitative was conducted as we described before^2^ after 9 days’ adipogenic differentiation with or without APR treatment. Related gene and protein expression level were also detected at the same time‐points.

### 2.4 Cell proliferation assay

Cell counting (CCK‐8 kit, Dojindo, Japan), cell cycle flow cytometry analysis (Cell Cycle Detection Kit, KeyGen Biotech, China) and EdU cell proliferation assay (iClick EdU Andy Fluor 488 Imaging Kit, GeneCopoeia, US) were performed to analyse the effect of APR on cell viability after 24 and 48 hours treatment for all investigated of cells. EdU‐positive cells were observed by fluorescent microscopy (Olympus, Tokyo, Japan) and related quantification was conducted by Image J software.

### 2.5 Cell migration assay

Transwell and wound‐healing assay were used for investigating the cell migration ability of BMSCs and ADSCs. For Transwell assay, cells were plated on top chamber overnight, then the medium of top chamber was changed to serum‐free medium and the bottom was changed to 10% foetal bovine serum (FBS) medium with or without APR. 18 hours later, inserts were removed and fixed 4% formaldehyde. The upper layer cells were removed by sterile cotton, and the bottom layer cells were stained with 0.5% crystal violet and counted with five fields under microscope. For wound‐healing assay, 200 µL pipette tip was used to make a scratch when the cells reached 100% confluence. Before and after 18 hours’ serum‐free treatment with or without APR, particular points of the scratch were photographed, and the scratch area was measured by Image J software.

### 2.6 Mice bone defect model and APR treatment

All the animal investigations were conducted according to the approved protocol by Animal Research Committee of Sichuan University (Chengdu, China) and in consistent with related guidelines and regulations. In total, 24 6‐week‐old male mice were used in this study, 8 mice per group. After anaesthesia, a monolayer‐cortical bone defect (1 mm in diameter) was generated around 1 mm below the growth plate in the distal side of femur using a dental engine (Wallingford, USA). APR diluted in corn oil was orally gavaged (5 mg/kg and 50 mg/kg body weight) every day after surgery; control group was fed with equal volume of corn oil.

8 mice from each group were sacrificed at 1 week after APR treatment, inguinal adipose tissue was isolated and preserved in −80 after snap frozen by liquid nitrogen. Femur defect samples and other organs including heart, kidney and liver were fixed in 4% paraformaldehyde solution for 6 hours and then changed to 70% ethanol.

### 2.7 Micro‐CT scanning and histology staining

8 femur samples/group from post‐treatment mice were scanned and analysed by micro‐CT (u‐CT80, SCANCO, Switzerland) as described before.^2^ 1 mm thickness femur bone defect (diameter: 1 mm) from bone surface was chosen as the region of interest. Several bone‐related measurement including bone volume to total volume ratio (BV/TV), trabecular separation (Tb.Sp), trabecular number (Tb.N) and trabecular thickness (Tb.Th) was evaluated.

After micro‐CT analysis, the femur samples were used for further histological staining as we previously reported.^2^ Bone tissue sections were stained with Masson and H&E staining. Adipose tissue frozen sections were stained with H&E, ORO staining and anti‐lipoprotein lipase immunofluorescence staining. Adipocyte size and adipocyte number in inguinal fat tissues were measured by Image J software. Heart, kidney and liver samples were stained with H&E staining to evaluate potential drug toxicity.

### 2.8 RNA extraction, RT‐PCR analysis and immunoblotting

TRIzol (Takara, Japan) was used for total RNA extraction from the treated cells or in vivo adipose tissues; RT‐PCR was performed according to the instructions as we described before.^2^ Primers used for RT‐PCR are listed in Table S1. Immunoblots were performed as we previously described.^2^ Treated cell samples were lysed with RIPA lysis buffer for around 30 min on ice. Antibodies used for immunoblot were listed in Table S2. Related quantitative of immunoblots was conducted via Image J software.

### 2.9 Statistical analysis

All quantitative data are shown as mean ± standard deviation. Statistical significance was assessed with one‐way analysis of variance (ANOVA) following Dunnett's test among 3 or more groups using SPSS software (version: 17.0) and Prism GraphPad (version: 7.0). Values of *P* < .05 were considered statistically different.

## 3 RESULTS

### 3.1 The expression of AdipoR1 and AdipoR2, and characterization of BMSCs and ADSCs

The expression of AR1 and AR2 was detected via immunofluorescence in MC3T3‐E1, RAW264.7, BMSCs and ADSCs (Figure S1A). The results showed that both AR1 and AR2 were uniformly distributed in the cytoplasm and cytomembrane in all cell types.

Third passage ADSCs and BMSCs were used for flow cytometry. Flow cytometry results indicated that rat ADSCs were positive for CD44 (97.4%) and CD29 (98.9%) but negative for CD45 (5.87%) and CD31 (5.08%) (Figure S1B). Flow cytometry results for rat BMSCs also showed two positive including CD29 (99.6%), CD90 (99.6%) and two negative markers including CD34 (0.71%) and CD45 (1.00%) (Figure S1C).

### 3.2 APR inhibited the proliferation of MC3T3‐E1 and RAW264.7

**FIGURE 1:**
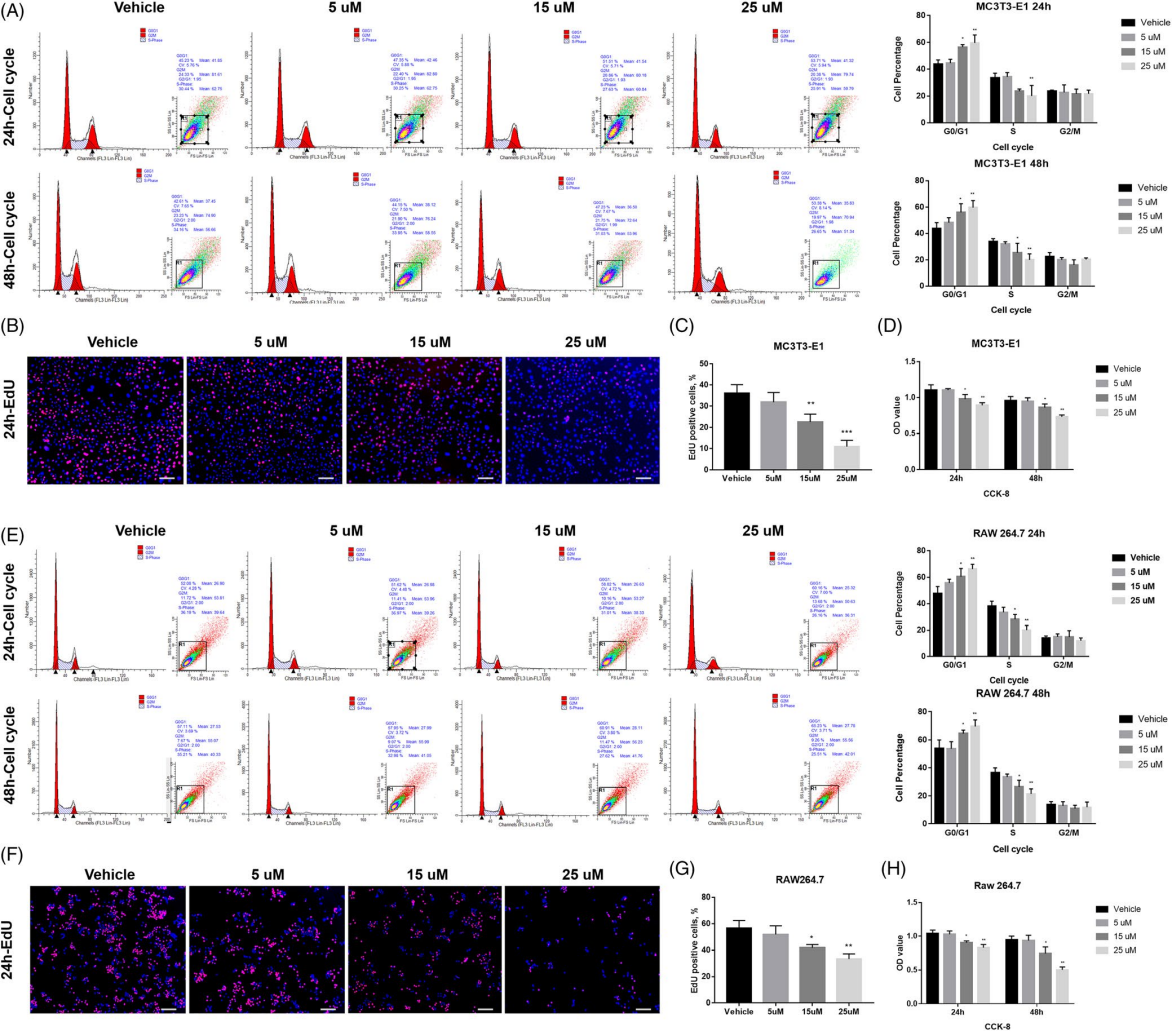
APR inhibited the proliferation of MC3T3‐E1 and RAW264.7. A, Cell cycle/apoptosis flow cytometry analysis and related quantitative analysis of MC3T3‐E1 after 24‐h and 48‐hour APR treatment, n = 3. B, Representative pictures for EdU staining of MC3T3‐E1 after 24‐h APR treatment and 2‐h EdU incubation, white scale bar: 250 µm. C, Quantification analysis of EdU assay for MC3T3‐E1 cells after 24‐h APR treatment and 2‐h EdU incubation, n = 3. D, CCK‐8 assay of MC3T3‐E1 after 24‐h and 48‐hour APR treatment, n = 5. E, Cell cycle/apoptosis flow cytometry analysis and related quantitative analysis of RAW264.7 after 24‐h and 48‐hour APR treatment, n = 3. F, Representative pictures for EdU staining of RAW264.7 after 24‐h APR treatment and 2‐h EdU incubation, white scale bar: 200 µm. G, Quantification analysis of EdU assay for RAW264.7 cells after 24‐h APR treatment and 2‐h EdU incubation, n = 3. H, CCK‐8 assay of RAW264.7 after 24‐h and 48‐hour APR treatment, n = 5. Data shown as mean ± SD. *P<.05 vs Vehicle; **P<.01 vs Vehicle

Initially, we investigated the effect of APR on cell survival and cell cycle distribution of pre‐osteoblast MC3T3‐E1 and pre‐osteoclast RAW264.7. We found the proliferation of the both osteoblast and osteoclast progenitors, MC3T3‐E1 and RAW264.7, was inhibited by APR treatment (Figure 1). The results of CCK‐8 assay showed that 15 µM and 25 µM APR could significantly inhibit cell proliferation of MC3T3‐E1 and RAW264.7 cells after 24‐ and 48‐hour treatment (Figure 1D, H). Further cell cycle/apoptosis (Figure 1A, E) and EdU analysis (Figure 1B, F) indicated that there is no significant cell apoptosis, but the S phase cells were remarkably decreased with APR treatment and increasing cells were arrested in G1/G0 phase (Figure 1C, G).

### 3.3 APR has no effect on the proliferation of BMSCs and ADSCs, but promote cell migration

**FIGURE 2:**
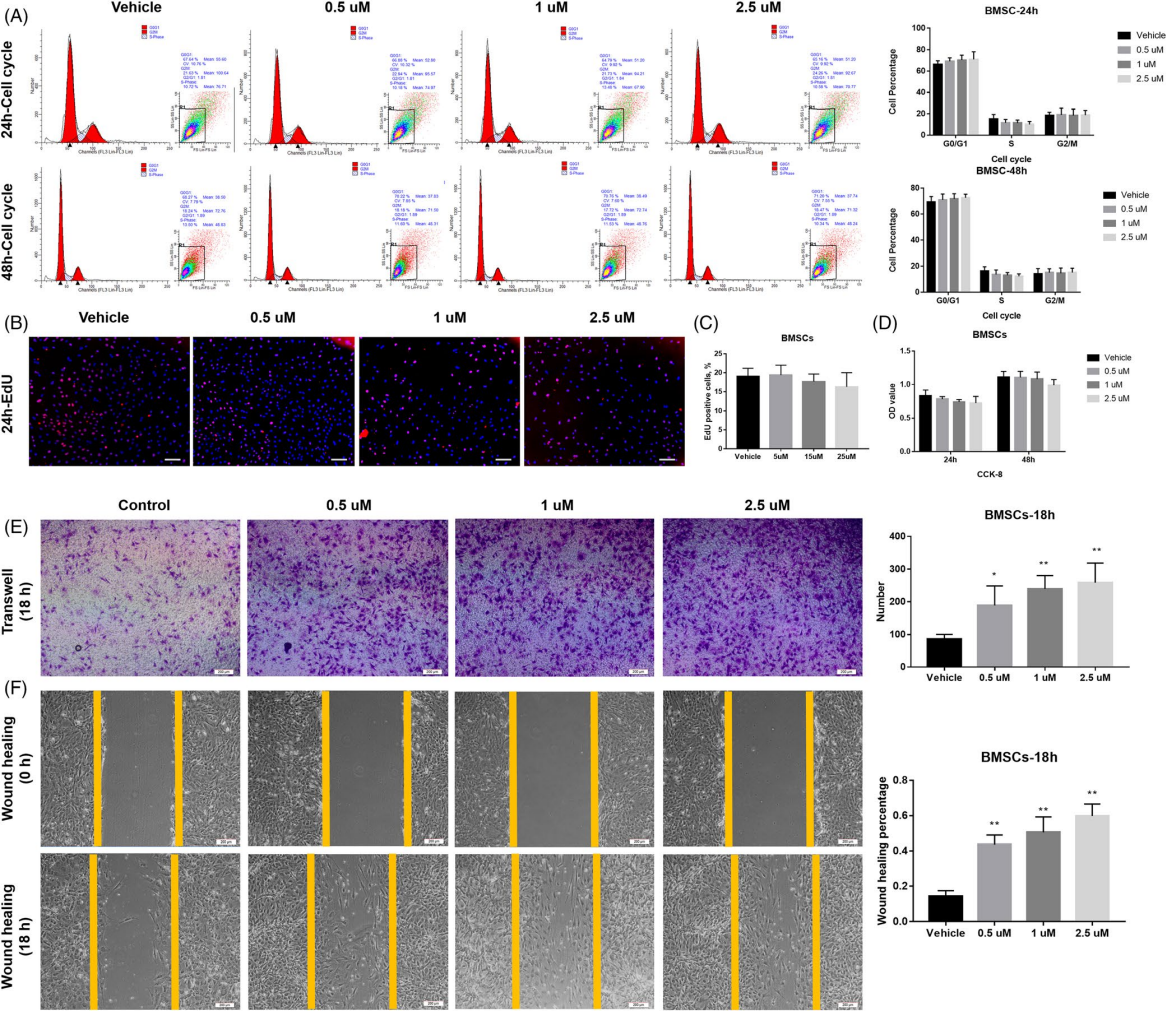
APR showed no effect on the proliferation of BMSCs but promote cell migration in a dose‐depend manner. A, Cell cycle/apoptosis flow cytometry analysis and related quantitative analysis of BMSCs after 24‐h and 48‐hour APR treatment, n = 3. B, Representative pictures for EdU staining of BMSCs after 24‐h APR treatment and 24‐h EdU incubation (with APR), white scale bar: 200 µm. C, Quantification analysis of EdU assay for rat BMSCs after 24‐h APR treatment and 24‐h EdU incubation, n = 3. D, CCK‐8 assay of BMSCs after 24‐h and 48‐hour APR treatment, n = 5. E, Representative images of Transwell assay for BMSCs after 18‐h APR treatment and related quantitative analysis with 5 random fields’ cell counting. F, Representative images of wound‐healing assay for BMSCs after 18‐h APR treatment under serum‐free condition and related quantitative analysis, n = 3. Data shown as mean ± SD. *P<.05 vs Vehicle; **P<.01 vs Vehicle

As indicated by our pre‐test, APR treatment that is higher than 5 µM significantly inhibited both cell proliferation and osteogenic/adipogenic differentiation of rat BSMCs and ADSC (data not shown), 0‐2.5 µM APR was used in our experiments for BSMCs and ADSCs. For one of the adipocyte and osteoblast lineages descendent progenitors‐BMSCs, according to CCK‐8, cell cycle/apoptosis and EdU analysis, no significant difference was found regarding both cell cycle distribution (Figure 2A‐C) and cell viability (Figure 2D) under 24‐ or 48‐hour APR treatment. However, Transwell and wound‐healing assay suggested that 18‐hour APR treatment could obviously increase cell migration of BMSCs with dose‐depend manner (0‐2.5 µM) (Figure 2E, F).

**FIGURE 3:**
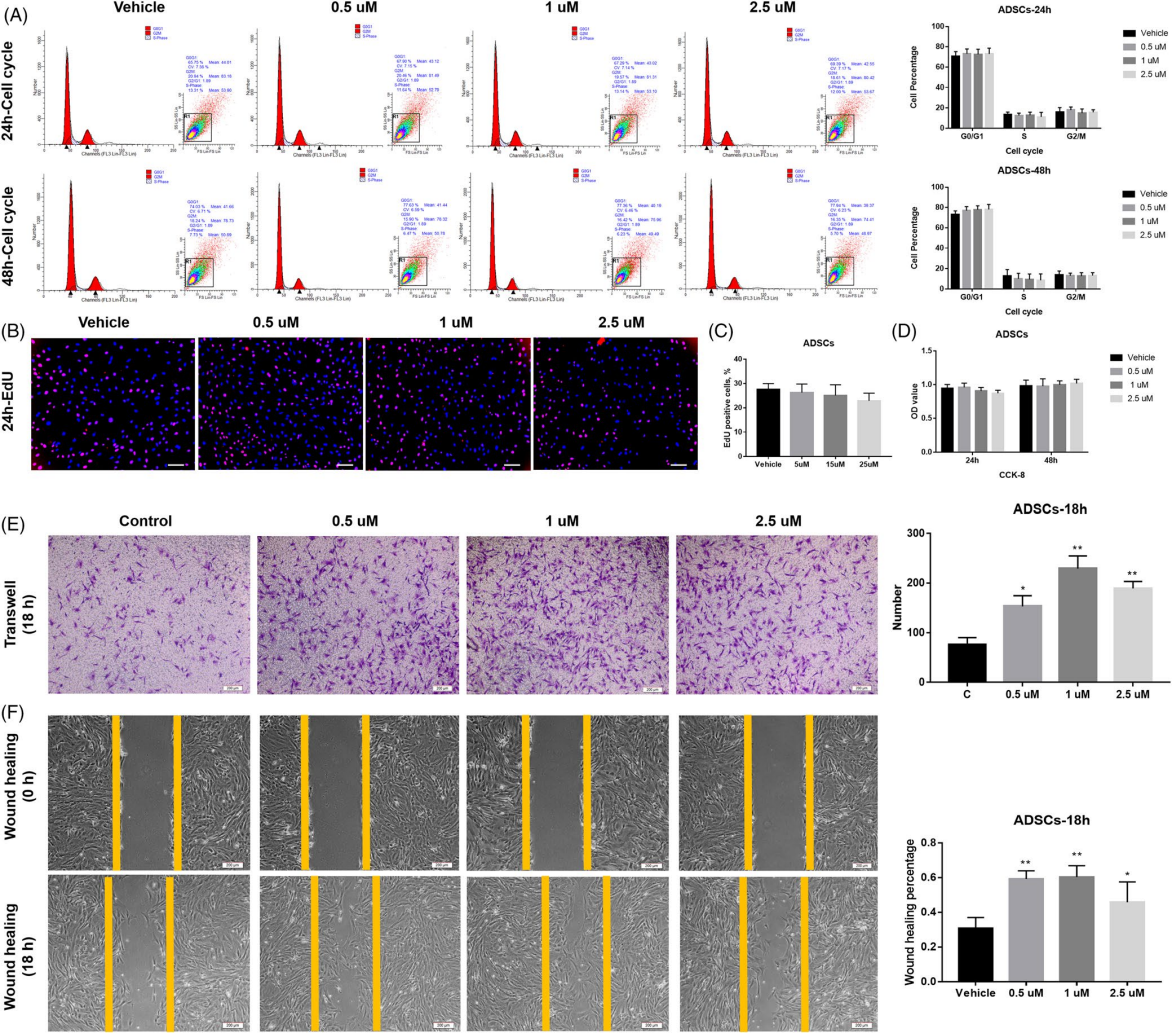
APR showed no effect on the proliferation of ADSCs but promote cell migration. A, Cell cycle/apoptosis flow cytometry analysis and related quantitative analysis of ADSCs after 24‐h and 48‐hour APR treatment, n = 3. B, Representative Pictures for EdU staining of ADSCs after 24‐h APR treatment and 24‐h EdU incubation (With APR), white scale bar: 200 µm. C, Quantification analysis of EdU assay for rat ADSCs after 24‐h APR treatment and 24‐h EdU incubation, n = 3. D, CCK‐8 assay of ADSCs after 24‐h and 48‐hour APR treatment, n = 5. E, Representative images of Transwell assay for ADSCs after 18‐h APR treatment and related quantitative analysis with 5 random fields’ cell counting. F, Representative images of wound‐healing assay for ADSCs after 18‐h APR treatment under serum‐free condition and related quantitative analysis, n = 3. Data shown as mean ± SD. *P<.05 vs Vehicle; **P<.01 vs Vehicle

For another adipocyte and osteoblast lineages progenitors‐ADSCs, APR showed similar effects to that on BMSCs, no difference was found for cell cycle distribution (Figure 3A‐C) and cell growth ability (Figure 3D). However, Transwell and wound‐healing assay suggested that 18‐hour APR treatment could obviously increase cell migration of both BMSCs and ADSCs (Figure 3E, F).

### 3.4 APR increased osteogenesis of MC3T3‐E1 and decreased osteoclastogenesis of RAW264.7

**FIGURE 4:**
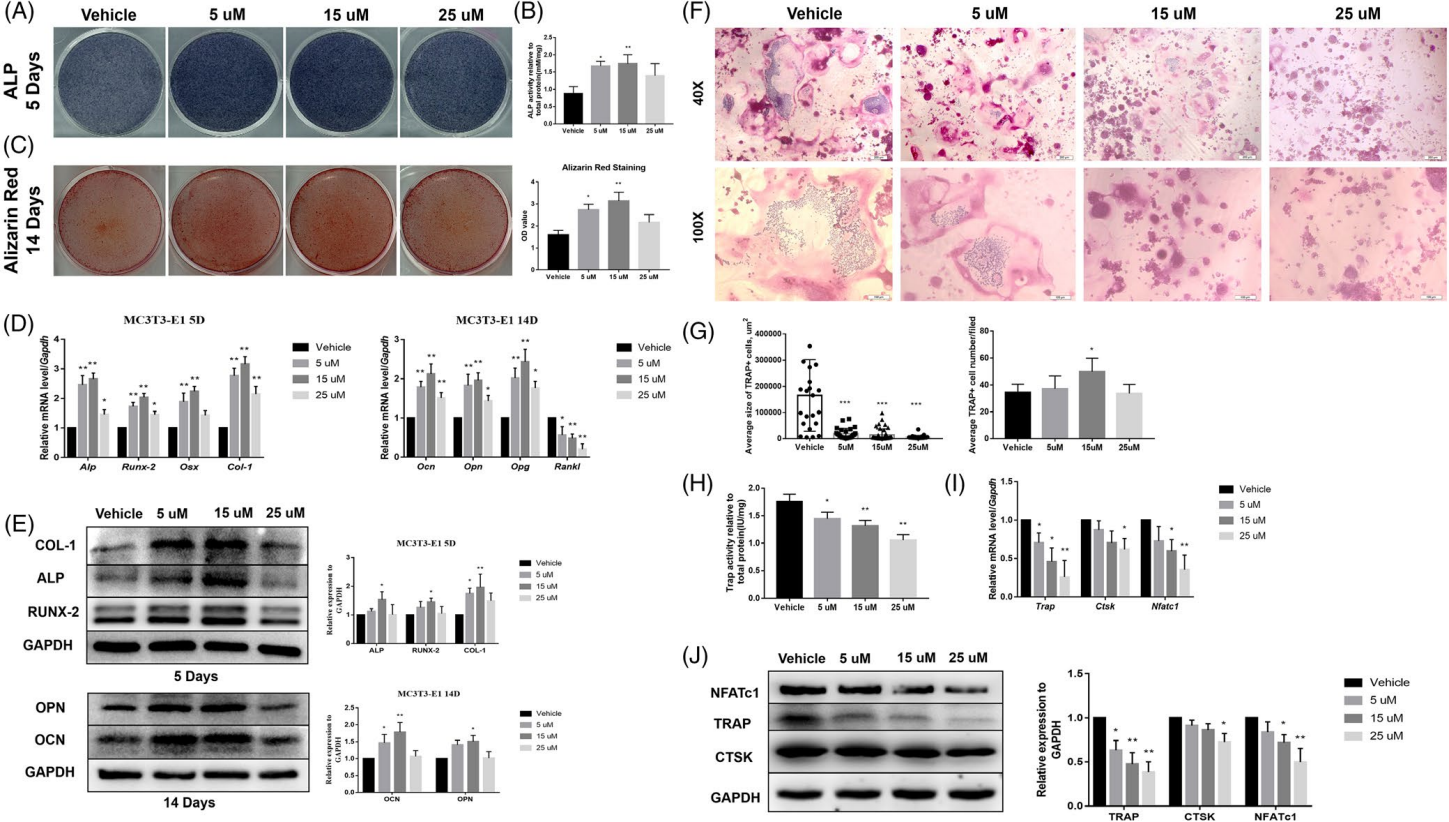
APR increase osteogenesis of MC3T3‐E1 and decrease osteoclastogenesis of RAW264.7. A, Representative images of ALP staining of MC3T3‐E1 after 5 days’ OB differentiation and APR treatment. B, ALP quantitative analysis of MC3T3‐E1 after 5 days’ OB differentiation and APR treatment, n = 3. C, Representative images of Alizarin Red of MC3T3‐E1 after 14 days’ OB differentiation and APR treatment. D, qPCR results of MC3T3‐E1 after 5 and 14 days’ OB differentiation and APR treatment, n = 3. E, Western blot results of MC3T3‐E1 after 5 and 14 days’ OB differentiation and APR treatment, n = 3. F, Representative images of TRAP staining of RAW264.7 after 6 days’ OC differentiation and APR treatment. G, Quantification analysis for the size and number of TRAP + osteoclasts after 6 days’ OC differentiation and APR treatment, n = 5 fields. H, TRAP quantitative analysis of RAW264.7 after 6 days’ OC differentiation and APR treatment, n = 3. I, qPCR results of RAW264.7 after 6 days’ OC differentiation and APR treatment, n = 3. J, Western blot results of RAW264.7 after 6 days’ OC differentiation and APR treatment, n = 3. Data shown as mean ± SD. *P < .05 vs Vehicle; **P < .01 vs Vehicle

Subsequently, we further investigated the effect of APR on bone‐fat balance including osteoblast‐osteoclast and osteoblast‐adipocyte differentiation balance. With continuous APR treatment along OB differentiation, Alkaline Phosphatase (ALP) staining results of MC3T3‐E1 showed that 5 and 15 µM group had significantly higher ALP activity after 3‐7 days’ OB differentiation while 25 µM showed no difference compared to vehicle group (Figure 4A, B; Figure S2A, B). Similarly, Alizarin Red staining showed the same trend after 14 days of OB differentiation (Figure 4C). Besides, qPCR results also showed that the gene expression of *Alp*, *Runx‐2*, *Osx* and *Col‐1* was significantly upregulated by 5 µM and 15 µM APR treatment with 3‐7 days’ OB differentiation, and the gene expression level of *Ocn, Opn, Opg* was also increased with 14 days’ 5 µM and 15 µM APR treatment (Figure 4D; Figure S2C). Not surprisingly, the upregulation of these OB‐related genes declined at 25 µM. And it is noteworthy that the expression of *Rankl* is decreased with APR treatment in a dose‐dependent manner (Figure 4D). Western blot results further confirmed these findings (Figure 4E).

Additionally, TRAP staining results suggested a dose‐dependent inhibition of osteoclast formation by treating RAW264.7 with APR (Figure 4F‐H). Although significant decreased size of osteoclasts was found in AdipoRon‐treated groups, there is no difference for the number of osteoclasts among groups except 15 µM AdipoRon group. qPCR and Western blot results further suggested that the gene and protein expression level of OC related transcript factors and functional proteins including CTSK, TRAP and NFATc1 were significantly decreased in APR treatment groups (Figure 4I, J).

### 3.5 APR showed pro‐osteogenic and anti‐adipogenic effect on the rat BMSCs and ADSCs

**FIGURE 5:**
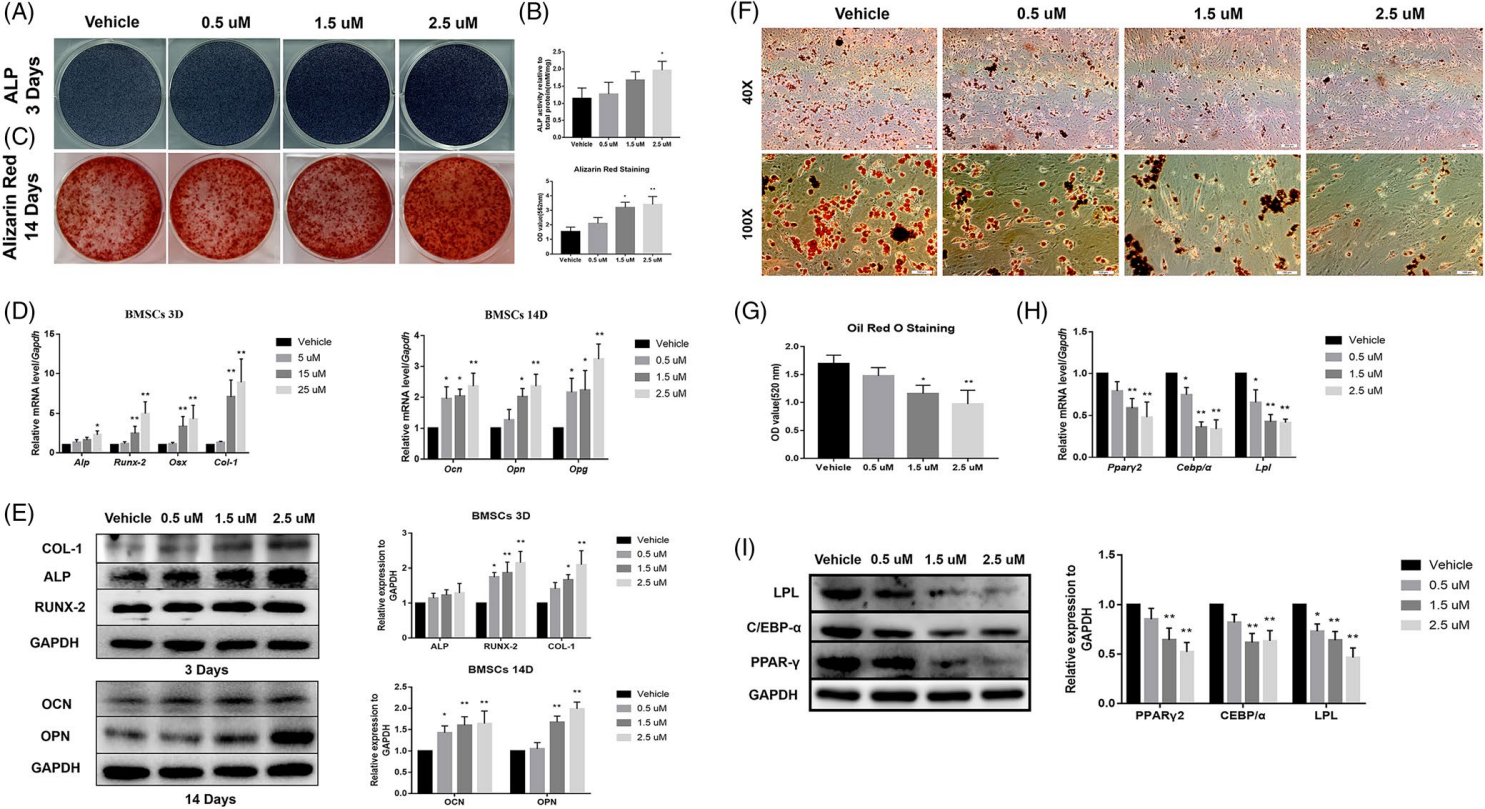
APR showed pro‐osteogenic and anti‐adipogenic effect on rat BMSCs. A, Representative images of ALP staining of BMSCs after 3 days’ OB differentiation and APR treatment. B, ALP quantitative analysis of BMSCs after 3 days’ OB differentiation and APR treatment, n = 3. C, Representative images of Alizarin Red of BMSCs after 14 days’ OB differentiation and APR treatment. D, qPCR results of BMSCs after 3 and 14 days’ OB differentiation and APR treatment, n = 3. E, Western blot results of BMSCs after 3 and 14 days’ OB differentiation and APR treatment, n = 3. F, Representative images of ORO staining of BMSCs after 9 days’ AD differentiation and APR treatment. G, ORO quantitative analysis of BMSCs after 9 days’ AD differentiation and APR treatment, n = 3. H, qPCR results of BMSCs after 9 days’ AD differentiation and APR treatment, n = 3. I, Western blot results of BMSCs after 9 days’ AD differentiation and APR treatment, n = 3. Data shown as mean ± SD. *P < .05 vs Vehicle; **P < .01 vs Vehicle

In this part, 4‐week‐old male SD rats were used for BMSCs and ADSCs isolation and further experiments. For BMSCs, ALP and Alizarin Red staining results showed that continuously APR treatment along with OB differentiation leads to significantly increased ALP activity (3‐7 days) and mineralization formation (14 days) (Figure 5A‐C; Figure S3A, B). qPCR results showed that 1.5 µM and 2.5 µM APR could remarkably upregulate the gene expression of *Alp, Runx2, Osx* and *Col‐1* with 3‐7 days’ OB differentiation and increase the gene expression of *Ocn, Opn* and *Opg* with 14 days’ OB differentiation (Figure 5D; Figure S3C). Western blot results further confirmed qPCR results (Figure 5E). Moreover, ORO staining and related quantitative results indicated that the adipogenesis of BMSCs was inhibited by APR treatment with 9 days’ AD differentiation (Figure 5F, g). Consistently, the expression level of AD differentiation‐related genes and proteins including PPARγ2, CEBP/α and LPL was found significantly down‐regulated in 1.5 µM and 2.5 µM APR group compared to vehicle group (Figure 5H, I).

**FIGURE 6:**
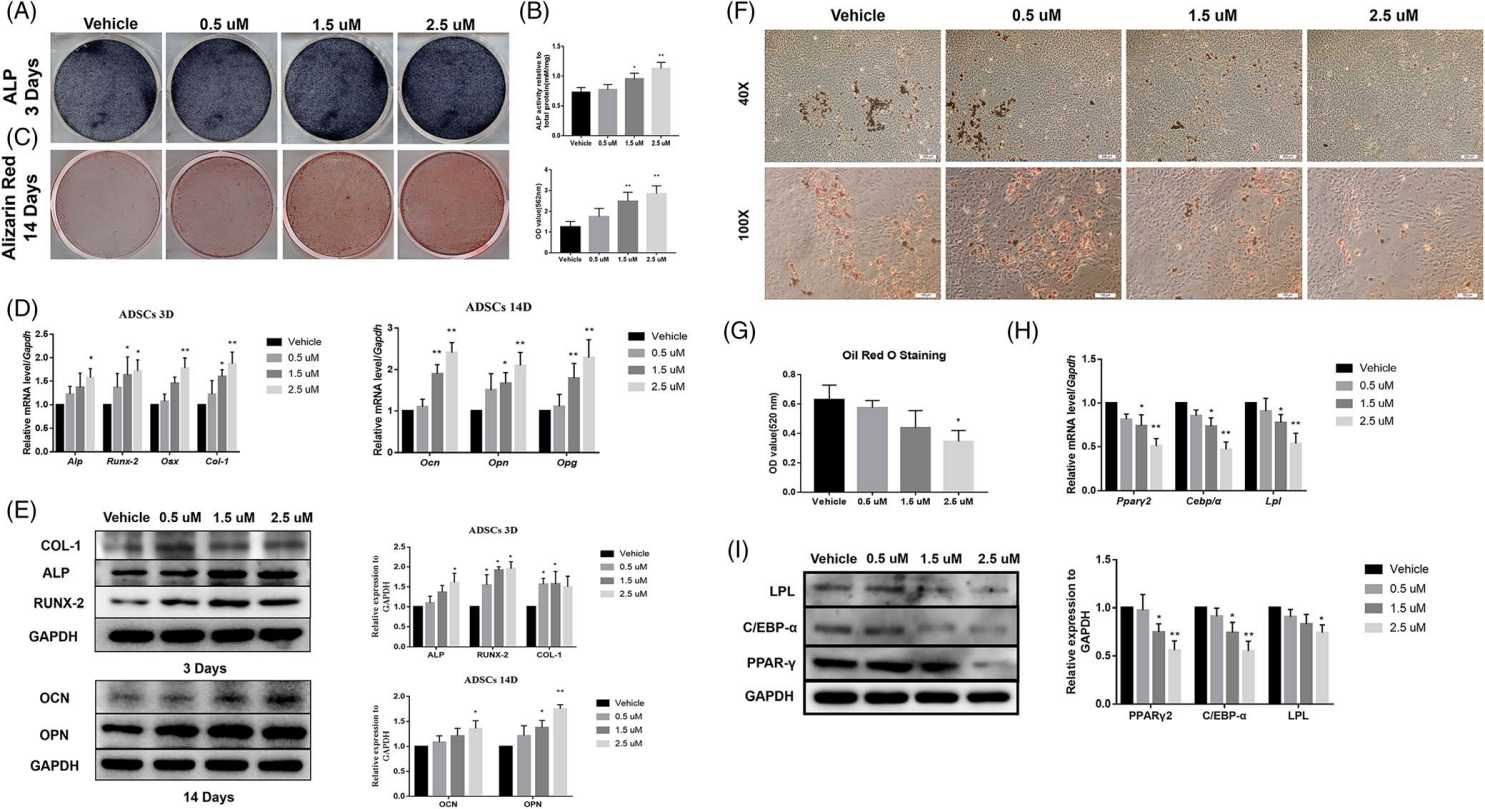
APR showed pro‐osteogenic and anti‐adipogenic effect on rat ADSCs. A, Representative images of ALP staining of ADSCs after 3 days’ OB differentiation and APR treatment. B, ALP quantitative analysis of ADSCs after 3 days’ OB differentiation and APR treatment, n = 3. C, Representative images of Alizarin Red of ADSCs after 14 days’ OB differentiation and APR treatment. D, qPCR results of ADSCs after 3 and 14 days’ OB differentiation and APR treatment, n = 3. E, Western blot results of ADSCs after 3 and 14 days’ OB differentiation and APR treatment, n = 3. F, Representative images of ORO staining of ADSCs after 9 days’ AD differentiation and APR treatment. G, ORO quantitative analysis of ADSCs after 9 days’ AD differentiation and APR treatment, n = 3. H, qPCR results of ADSCs after 9 days’ AD differentiation and APR treatment, n = 3. I, Western blot results of ADSCs after 9 days’ AD differentiation and APR treatment, n = 3. Data shown as mean ± SD. *P < .05 vs Vehicle; **P < .01 vs Vehicle

Furthermore, we also found that APR has similar but limited pro‐osteogenic effect on rat ADSCs. APR could also increase ALP activity and promote mineralization formation at 1.5 µM and 2.5 µM in ADSCs with OB differentiation (Figure 6A‐C; Figure S4A, B); meanwhile, the expression level of OB‐related genes and proteins was upregulated in 1.5 µM and especially 2.5 µM APR group (Figure 6D, E; Figure S1C). Not surprisingly, 9 days’ APR treatment with AD differentiation strongly inhibited adipocyte formation (Figure 6F, G) and the expression of PPARγ2, CEBP/α and LPL (Figure 6 H, I) in ADSCs.

### 3.6 APR promoted femur defect repairing and inhibited inguinal fat accumulation

**FIGURE 7:**
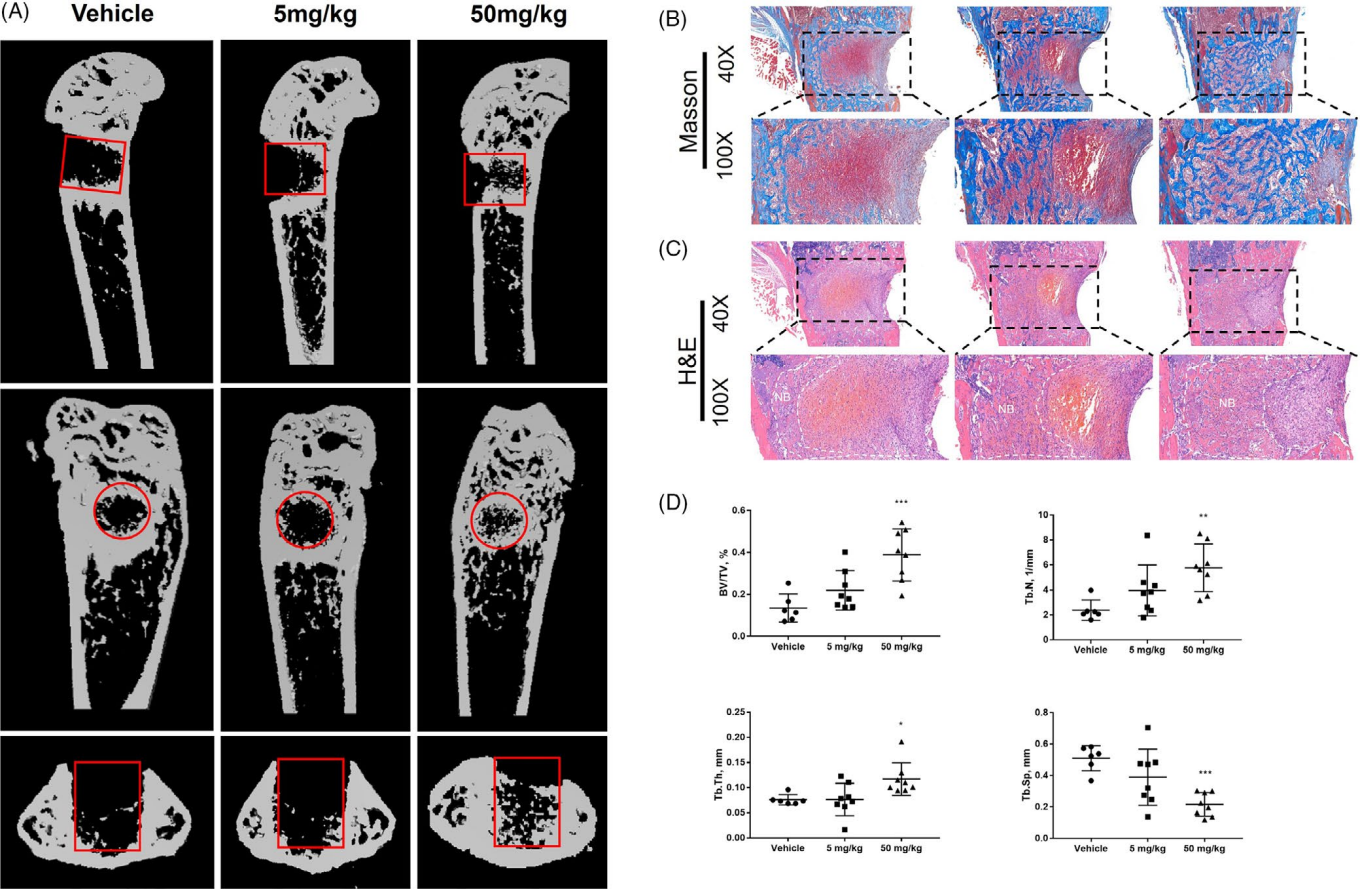
APR promoted femur defect repairing in male mice. A, Representative images of micro‐CT of femur at 1 week after surgery, red cycle/rectangular: bone defect region. B, Representative Masson staining pictures of femur at 1 week after surgery. C, Representative H&E pictures of femur at 1 week after surgery, NB: new bone. D, Quantitative analysis of femur micro‐CT results at 1 week after surgery, n = 6‐8. Data shown as mean ± SD. *P < .05 vs Vehicle; **P < .01 vs Vehicle

Based on the in vitro effect of APR mentioned above, we conducted animal studies to verify these findings in vivo using 6‐week‐old male mice. After bone defect surgery, mice were gavage fed with or without APR every day for 1 week. In the femur defect mice model, micro‐CT data showed significantly increased trabecular volume, thickness and number and decreased trabecular separation was observed in 50 mg/kg APR treatment group but not 5 mg/kg group compared to vehicle group at 1 week after surgery (Figure 7A, D). H&E and Masson staining analysis showed the same findings as micro‐CT: more newly formed trabecular bone was found in APR groups after 1‐week treatment (Figure 7B, C).

**FIGURE 8:**
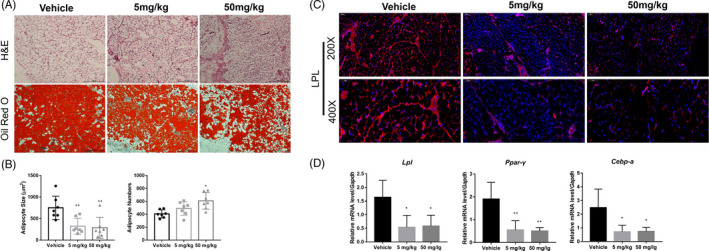
APR inhibited adipogenesis in inguinal fat pad of male mice. A, Representative H&E and Oil Red O staining pictures of inguinal adipocytes at 1 week after surgery. B, Quantitative analysis of adipocyte size and number in inguinal fat after 1‐week APR treatment, n = 7. C, Representative LPL‐immunofluorescence pictures of inguinal adipocytes at 1‐week post‐treatment. D, qPCR results of *Lpl, Ppar‐γ, Cebp‐α* in inguinal fat after 1‐week APR treatment, n = 7. Data shown as mean ± SD. *P < .05 vs Vehicle; **P < .01 vs Vehicle

Additionally, the inguinal fat pad was also isolated for investigating the effect of APR on adipogenesis after 1‐week treatment. H&E and ORO staining results indicated that the adipocytes were smaller in 5 mg/kg and 50 mg/kg group compared to vehicle group, but 50 mg/kg group seemed has slightly higher adipocyte number (Figure 8A, B). Immunofluorescence also showed LPL‐positive cells were remarkably decreased in APR groups after 1‐week ARP treatment (Figure 8C); qPCR results further confirmed that the expression level of adipogenesis‐related genes including *Lpl, Ppar‐γ* and *Cebp‐α* was significantly inhibited in APR groups (Figure 8D).

No significant drug toxicity‐related injury was found in heart, kidney and liver samples (Figure S5).

## 4 DISCUSSION

Growing evidence indicated that APN could be a promising therapeutic target in osteoporosis due to that it might be a determinant of lineage allocation in bone and bone marrow niche. However, regarding the diverse effect of APN on bone metabolism resulting from complicated endocrine/paracrine system and various APN isoforms, it would be hard to predict the efficacy of APN in clinical application. Besides, the third receptor of APN, T‐cadherin, was considered to be the receptor of native serum APN and play an important role in myogenesis and angiogenesis^21^, ^22^; these studies suggested that molecular weight (HWM) APN could directly bind to T‐cadherin, while AR1 and AR2 were activated by low molecular weight (LMW) forms APN. This might give some clues for the diverse effect of APN on bone metabolism that T‐cadherin might also serves as a receptor in bone niches. Hence, it would necessary to investigate the specific role for each adiponectin receptor during bone regeneration to comprehensively understand the effect of APN in bone metabolism. In our study, we focused on AR1 and AR2 signalling, and we used a specific activator for AR1 and AR2, AdipoRon, to treat pre‐osteoblast, pre‐osteoclast and mesenchymal stem cells in order to study how does ARs signalling regulate cell proliferation, stem cell migration, osteoblastgenesis, adipogenesis and osteoclastogenesis.

In our study, we first demonstrated that APR could slightly inhibit cell proliferation of both osteoblast and osteoclast progenitors—MC3T3‐E1 and RAW264.7 via arresting G1/G0 phase cells and decreasing S phase cells. In addition, APR showed no effect on the proliferation or cell cycle distribution of rat bone marrow mesenchymal cells and adipocyte‐derived mesenchymal cells, but APR significantly promoted cell migration of them. The results were consistent with previous study that the migration ability of mesenchymal cells (MSCs) could be enhanced by APR potentially via activating COX‐2/PGE2/HIF‐1 pathway.^17^ Moreover, we further investigated the effect of APR on bone‐fat cross‐talk including osteogenesis‐osteoclastogenesis balance and osteogenesis‐adipogenesis balance. Our results suggested that APR could promote osteogenic differentiation of both osteoblast‐precursor cells and MSCs, inhibit osteoclast differentiation from osteoclast‐precursor cells and decrease adipogenesis of MSCs. Consistently, adipogenesis in C3H10T1/2 cells was also inhibited with APR treatment by promoting the phosphorylation of AMPK and ACC.^23^ Meanwhile, the gene expression of *Rank ligand* was found decreased while *Opg* was found increased in osteoblast precursors. Similar results were found previously that APR treatment could significantly inhibit osteoclastogenesis of RAW 264.7 cells and bone marrow macrophages in dependent of cell proliferation.^19^ Our data showed that APR treated group had much smaller osteoclasts but similar osteoclast number, which indicated that the cell fusion was dramatically inhibited in the late stage. Interestingly, previous study showed that APN increased the formation and function of osteoclasts indirectly by promoting RANKL secretion and repressing OPG production from osteoblasts. Depending on our findings that ARs activation could directly downregulate the ratio between *Rankl* and *Opg*, the diverse effect might be resulted from the activation of the third receptor of APN, T‐cadherin.

Given that ARs signalling activation could improve bone‐fat imbalance and promote osteogenesis, inhibit osteoclastogenesis and adipogenesis, APR would be a promising drug for preventing diabetes‐related bone fractures and oestrogen deficiency‐induced osteoporosis. Similarly, Wu et al applied APR in mice diabetic periodontitis and found that APR treatment group had significantly lower osteoclast numbers and suppressed alveolar bone resorption.^19^ Therefore, based on the in vitro data and previous study, we hypothesized that APR could promote bone regeneration and defect healing. We then established femur defect model with 6‐week‐old male mice and evaluated the impact of APR in accelerating bone regeneration. We found much more new bone formation, higher BV/TV, increasing Tb.Th, Tb.N and lower Tb.Sp in APR treated mice compared to vehicle‐treated mice. Meanwhile, decreased adipocyte size of inguinal adipocytes and down‐regulated adipogenesis‐related genes expression including *Pparγ*, *Cebpaα* and *Lpl* in inguinal fat pad were found in APR treatment group, although 50 mg/kg group showed lightly higher adipocyte number. These data may indicate that the lipid accumulation was significantly inhibited but not the adipocyte formation. The protein expression level of LPL was also inhibited in APR treatment group. Our previous study showed APN level that secreted from inguinal adipose tissue was negatively related to bone regeneration in tibia defects.^2^ We believed that might be due to the changes of other circulating bone‐related hormones or APN feedback regulation instead of its own effect on bone‐fat cross‐talk. In this study, we further confirmed that the direct activation of ARs signalling promoted bone formation and defect healing.

In conclusion, our study demonstrated a positive role of ARs activation in regulating bone‐fat balance including pro‐osteogenesis, anti‐osteoclastogenesis and anti‐adipogenesis in young mice‐derived MSCs and osteoclast‐precursors. In addition, APR treatment could also faster bone regeneration and defect healing. Therefore, ARs signalling should be a promising target for the treatment and prevention of osteoporosis.

## CONFLICT OF INTEREST

None.

## AUTHOR CONTRIBUTIONS

Hanghang Liu: conceptualization, investigation, methodology, formal analysis, resources, validation, visualization, writing—original draft preparation; Shibo Liu: investigation, formal analysis, validation, visualization; Huanzhong Ji: investigation, methodology, validation; Qiucheng Zhao: investigation, methodology; Yao Liu: supervision, writing—review and editing; Pei Hu: supervision, writing—review and editing; En Luo: conceptualization, funding acquisition, supervision, writing—review and editing.

## Supporting information

Data S1

## Data Availability

The data that support the findings of this study are available from the corresponding author upon reasonable request.

## References

[1] Kadowaki T , Yamauchi T , Waki H , Iwabu M , Okada‐Iwabu M , Nakamura M . Adiponectin, adiponectin receptors, and epigenetic regulation of adipogenesis. Cold Spring Harb Symp Quant Biol. 2011;76:257‐265. 22492282 10.1101/sqb.2012.76.010587

[2] Yang S , Liu H , Liu Y , Liu L , Zhang W , Luo E . Effect of adiponectin secreted from adipose‐derived stem cells on bone‐fat balance and bone defect healing. J Tissue Eng Regen Med. 2019;13(11):2055‐2066. 31210408 10.1002/term.2915

[3] Chen T , Wu Y‐W , Lu H , Guo Y , Tang Z‐H . Adiponectin enhances osteogenic differentiation in human adipose‐derived stem cells by activating the APPL1‐AMPK signaling pathway. Biochem Biophys Res Commun. 2015;461(2):237‐242. 25892517 10.1016/j.bbrc.2015.03.168

[4] Liu X , Chen T , Wu Y , Tang Z . Role and mechanism of PTEN in adiponectin‐induced osteogenesis in human bone marrow mesenchymal stem cells. Biochem Biophys Res Commun. 2017;483(1):712‐717. 27986563 10.1016/j.bbrc.2016.12.076

[5] Luo X‐H , Guo L‐J , Yuan L‐Q , et al. Adiponectin stimulates human osteoblasts proliferation and differentiation via the MAPK signaling pathway. Exp Cell Res. 2005;309(1):99‐109. 15963981 10.1016/j.yexcr.2005.05.021

[6] Pu Y , Wu H , Lu S , et al. Adiponectin promotes human jaw bone marrow stem cell osteogenesis. J Dent Res. 2016;95(7):769‐775. 26961489 10.1177/0022034516636853

[7] Lin YY , Wu SC , Liu BH , Chen CY , Mersmann HJ , Ding ST . Adiponectin receptor 1 involves in regulating bone formation and osteoblast differentiation. FASEB J. 2012;26Experimental Biology 2012, EB. San Diego, CA United States. (var.pagings).

[8] Wang Q‐P , Li X‐P , Wang M , et al. Adiponectin exerts its negative effect on bone metabolism via OPG/RANKL pathway: an in vivo study. Endocrine. 2014;47(3):845‐853. 24627163 10.1007/s12020-014-0216-z

[9] China SP , Pal S , Chattopadhyay S , et al. Globular adiponectin reverses osteo‐sarcopenia and altered body composition in ovariectomized rats. Bone. 2017;105:75‐86. 28811200 10.1016/j.bone.2017.08.005

[10] Hu X‐F , Wang L , Lu Y‐Z , et al. Adiponectin improves the osteointegration of titanium implant under diabetic conditions by reversing mitochondrial dysfunction via the AMPK pathway in vivo and in vitro. Acta Biomater. 2017;61:233‐248. 28624657 10.1016/j.actbio.2017.06.020

[11] Abbott M , O'Carroll D , Wang L , Roth T , Nissenson R . Direct actions of adiponectin on mature osteoblasts may contribute to negative regulation of skeletal homeostasis. J Bone Miner Res. 2013;28:2013.

[12] Luo X‐H , Guo L‐J , Xie H , et al. Adiponectin stimulates RANKL and inhibits OPG expression in human osteoblasts through the MAPK signaling pathway. J Bone Miner Res. 2006;21(10):1648‐1656. 16995820 10.1359/jbmr.060707

[13] Cornish J , Musson D , Naot D . Does the adipokine, adiponectin, play a role in the coupling between fat and bone? J Bone Miner Res. 2017;31(Supplement 1): 10.1002/jbmr.3107

[14] Lee SW . Association between metabolic risks and bone mineral density in postmenopausal women. Climacteric. 2016;19:96.

[15] Ealey KN , Kaludjerovic J , Archer MC , Ward WE . Adiponectin is a negative regulator of bone mineral and bone strength in growing mice. Exp Biol Med (Maywood). 2008;233(12):1546‐1553. 18849538 10.3181/0806-RM-192

[16] Okada‐Iwabu M , Yamauchi T , Iwabu M , et al. A small‐molecule AdipoR agonist for type 2 diabetes and short life in obesity. Nature. 2013;503(7477):493‐499. 24172895 10.1038/nature12656

[17] Malih S , Saidijam M , Mansouri K , et al. Promigratory and proangiogenic effects of AdipoRon on bone marrow‐derived mesenchymal stem cells: an in vitro study. Biotechnol Lett. 2017;39(1):39‐44. 27627895 10.1007/s10529-016-2214-0

[18] Wang Z , Tang J , Li Y , et al. AdipoRon promotes diabetic fracture repair through endochondral ossification‐based bone repair by enhancing survival and differentiation of chondrocytes. Exp Cell Res. 2019;387:111757. 31838062 10.1016/j.yexcr.2019.111757 PMC7722537

[19] Wu X , Qiu W , Hu Z , et al. An adiponectin receptor agonist reduces type 2 diabetic periodontitis. J Dent Res. 2019;98(3):313‐321. 30626266 10.1177/0022034518818449 PMC6385350

[20] Luo EN , Hu J , Bao C , et al. Sustained release of adiponectin improves osteogenesis around hydroxyapatite implants by suppressing osteoclast activity in ovariectomized rabbits. Acta Biomater. 2012;8(2):734‐743. 22061107 10.1016/j.actbio.2011.10.029 PMC3264062

[21] Kita S , Fukuda S , Maeda N , Shimomura I . Native adiponectin in serum binds to mammalian cells expressing T‐cadherin, but not AdipoRs or calreticulin. Elife. 2019;8: 10.7554/eLife.48675 PMC6822988 31647413

[22] Fukuda S , Kita S , Obata Y , et al. The unique prodomain of T‐cadherin plays a key role in adiponectin binding with the essential extracellular cadherin repeats 1 and 2. J Biol Chem. 2017;292(19):7840‐7849. 28325833 10.1074/jbc.M117.780734 PMC5427265

[23] Wang SJ , Lu WY , Liu KY . Adiponectin receptor agonist AdipoRon suppresses adipogenesis in C3H10T1/2 cells through the adenosine monophosphate‐activated protein kinase signaling pathway. Mol Med Rep. 2017;16(5):7163‐7169. 28901521 10.3892/mmr.2017.7450

