# Supplementary material for: An adiponectin receptor agonist promote osteogenesis via regulating bone‐fat balance
Source: Cell Prolif. 2021 May 3;54(6):e13035. doi: 10.1111/cpr.13035 (PMC8168410; doi:10.1111/cpr.13035)
Supplement: Supplementary file 1 — Data S1 [file CPR-54-e13035-s001.docx]

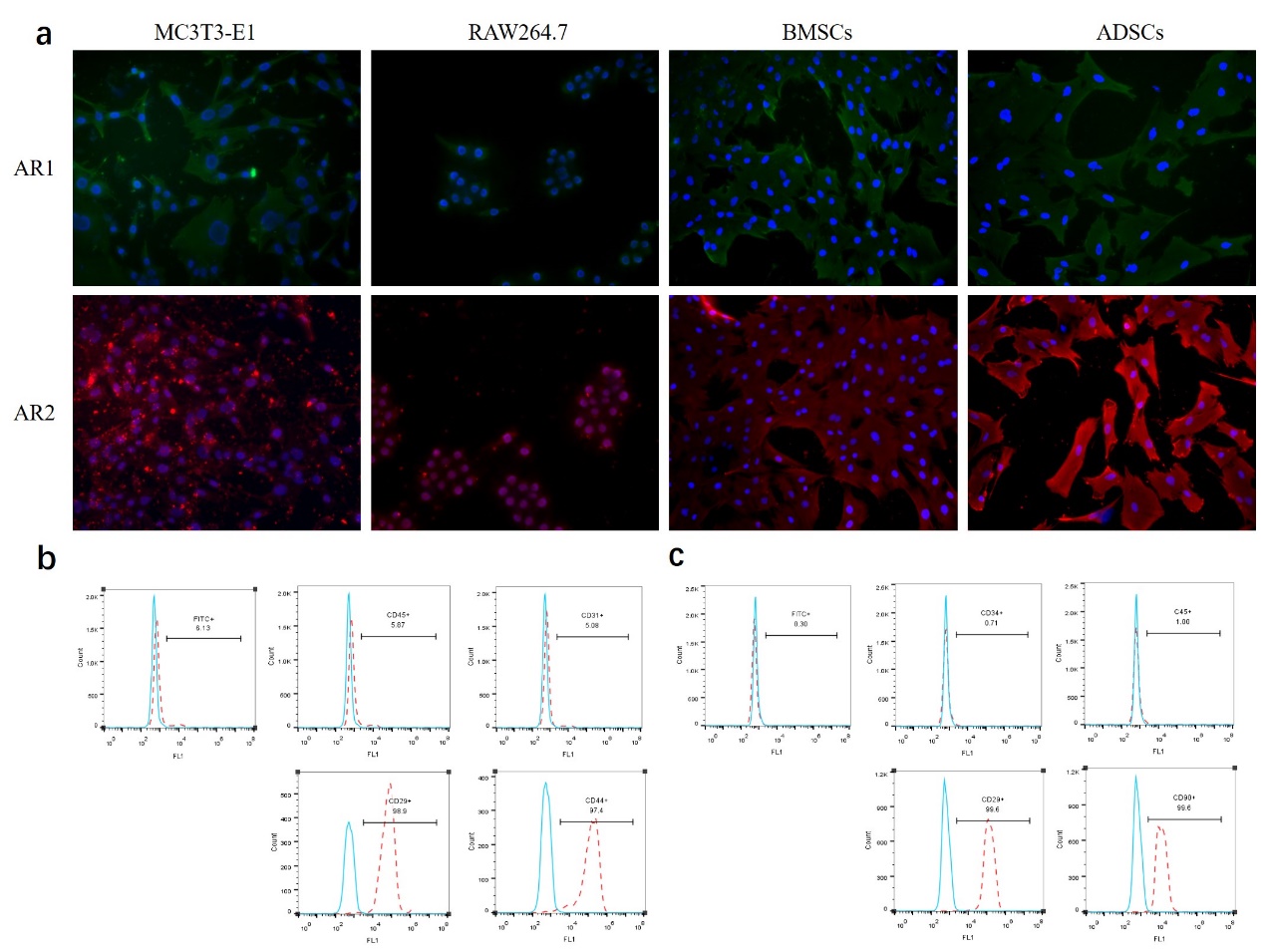


**Fig. S1**. The expression of AR1 and AR2, and characterization of BMSCs and ADSCs. **a** Immunofluorescence staining of AR1 and AR2 in MC3T3-E1, RAW264.7, BMSCs and ADSCs. **b** Histograms of ADSCs identification results by flow cytometry. **c** Histograms of the BMSCs identification results by flow cytometry.


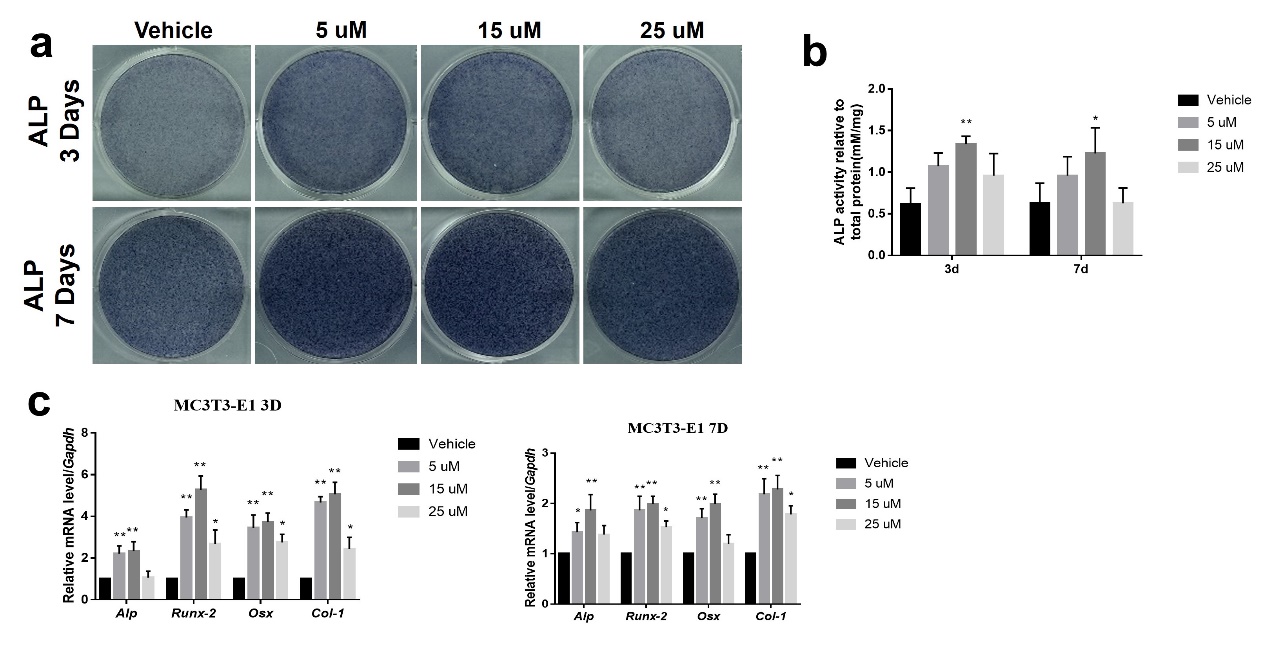


**Fig. S2** APR increase osteogenesis of MC3T3-E1. **a** Representative images of ALP staining of MC3T3-E1 after 3 and 7 days’ OB differentiation and APR treatment. **b** ALP quantitative analysis of MC3T3-E1 after 3 and 7 days’ OB differentiation and APR treatment, n=3. **c** qPCR results of MC3T3-E1 after 3 and 7 days’ OB differentiation and APR treatment, n=3. Data shown as mean ± SD. *p<.05 vs. Vehicle; **p<.01 vs. Vehicle.


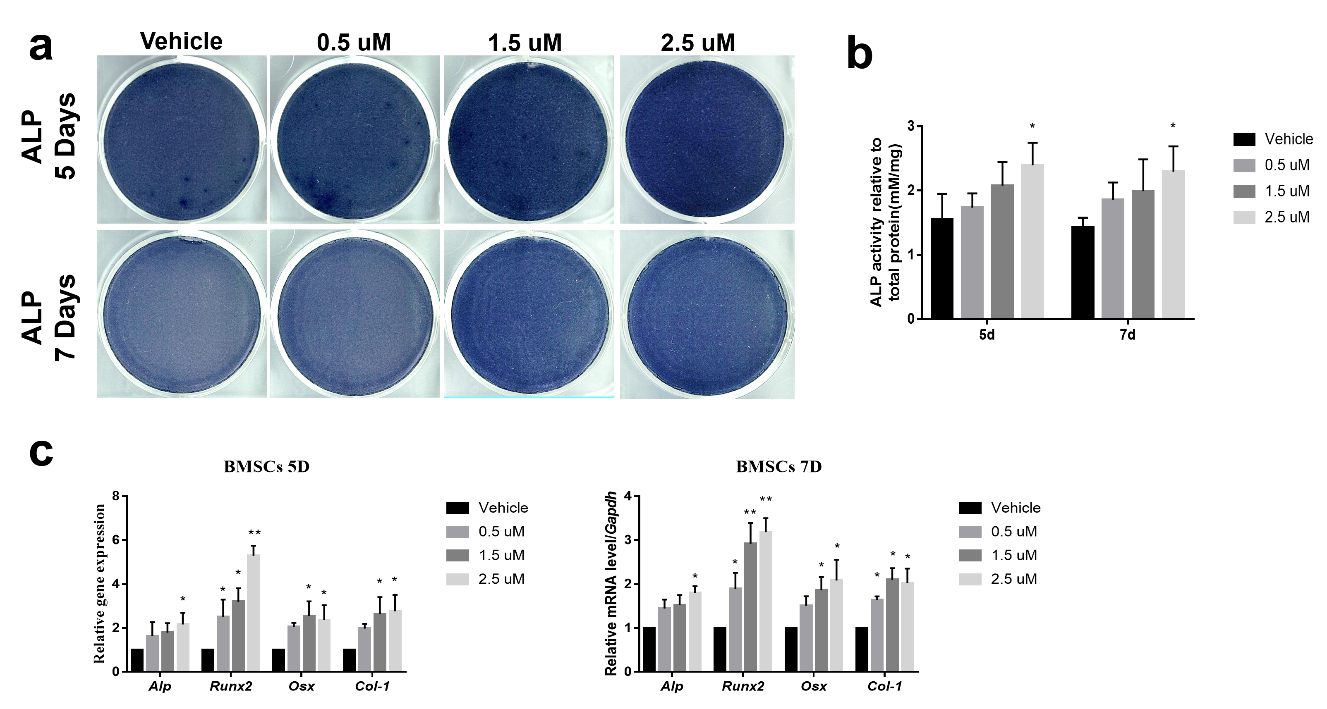


**Fig. S3** APR increase osteogenesis of rat BMSCs **a** Representative images of ALP staining of BMSCs after 5 and 7 days’ OB differentiation and APR treatment. **b** ALP quantitative analysis of BMSCs after 5 and 7 days’ OB differentiation and APR treatment, n=3. **c** qPCR results of BMSCs after 5 and 7 days’ OB differentiation and APR treatment, n=3. Data shown as mean ± SD. *p<.05 vs. Vehicle; **p<.01 vs. Vehicle.


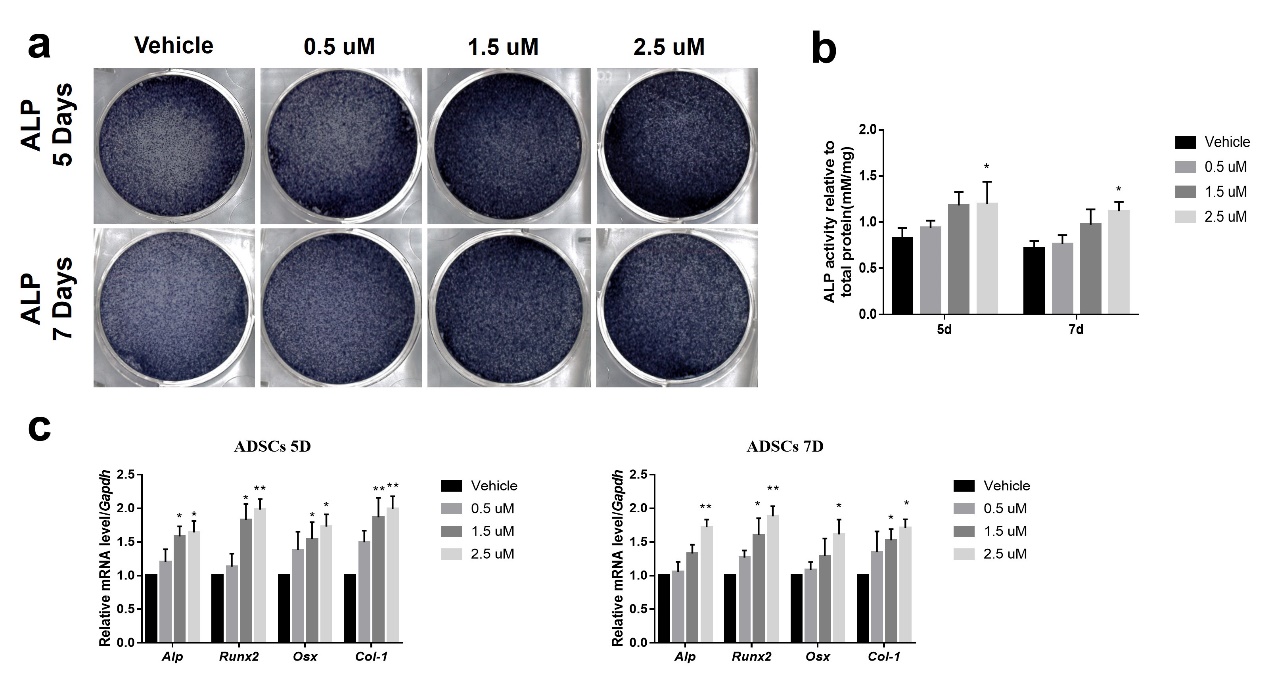


**Fig. S4** APR increase osteogenesis of rat ADSCs **a** Representative images of ALP staining of ADSCs after 5 and 7 days’ OB differentiation and APR treatment. **b** ALP quantitative analysis of ADSCs after 5 and 7 days’ OB differentiation and APR treatment, n=3. **c** qPCR results of ADSCs after 5 and 7 days’ OB differentiation and APR treatment, n=3. Data shown as mean ± SD. *p<.05 vs. Vehicle; **p<.01 vs. Vehicle.


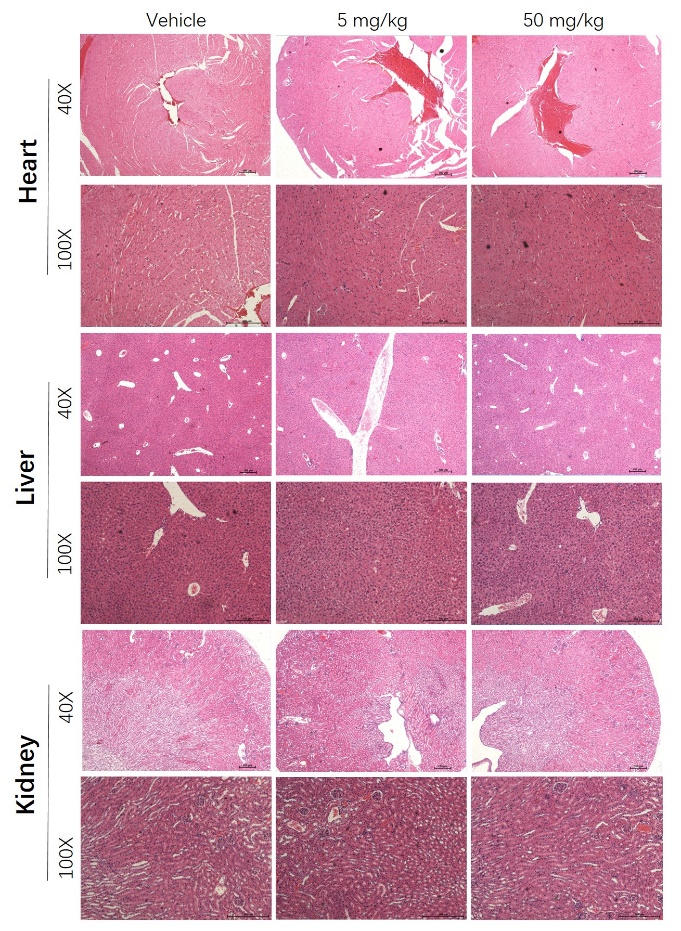


**Fig. S5** Representative HE staining images of heart, liver and kidney at 1-week post-treatment.

| **Gene** | **Forward Primer (5’-3’)** | **Reverse Primer (5’-3’)** |
| --- | --- | --- |
| Mice | | |
| *Gapdh* | AGGTCGGTGTGAACGGATTTG | TGTAGACCATGTAGTTGAGGTCA |
| *Alp* | GACTGGTACTCGGATAACGA | TGCGGTTCCAGACATAGTGG |
| *Runx-2* | CCAACCGAGTCATTTAAGGCT | GCTCACGTCGCTCATCTTG |
| *Osx/Sp7* | ATGGCGTCCTCTCTGCTTG | TGAAAGGTCAGCGTATGGCTT |
| *Col-1* | GGTGAGCCTGGTCAAACGG | ACTGTGTCCTTTCACGCCTTT |
| *Ocn* | CAAGTCCCACACAGCAGCTT | AAAGCCGAGCTGCCAGAGTT |
| *Opn* | CAGGGAGGCAGTGACTCTTC | AGTGTGGAAAGTGTGGCGTT |
| *Opg* | CAGAGAAGCCACGCAAAAGTG | AGCTGTGTCTCCGTTTTATCCT |
| *Rankl* | CGCCAACATTTGCTTTCGG | TTTTAACGACATACACCATCAGC |
| *Trap* | CACTCCCACCCTGAGATTTGT | CATCGTCTGCACGGTTCTG |
| *Ctsk* | AATACCTCCCTCTCGATCCTACA | TGGTTCTTGACTGGAGTAACGTA |
| *Nfatc1* | GGAGAGTCCGAGAATCGAGAT | TTGCAGCTAGGAAGTACGTCT |
| Rat | | |
| *Gapdh* | AGGAGTCCCCATCCCAACTC | ATAACCCCCACAACACTGCAT |
| *Alp* | CTGCCTCTCAGCTTCAGATCG | TGTCCTGTCCCATTCACACTG |
| *Runx-2* | AACCAAGTGGCCAGGTTCAA | GGTGGGGAGGATTGTGTCTG |
| *Osx/Sp7* | TTGAATGGTGAGGTGCAGACC | ATCACCAGCTAGCGTCCCTT |
| *Col-1* | GATGGACTCAACGGTCTCCC | CGGCCACCATCTTGAGACTT |
| *Ocn* | AACAGATTGTTGGGGCACAAG | CCCAGGCCCCTCAAACAATAA |
| *Opn* | AGTTCTCCTGGCTGAATTCTGAG | TTGCTGTAATGCGCCTTCTC |
| *Opg* | CAGAGAAGCCACGCAAAAGTG | AGCTGTGTCTCCGTTTTATCCT |
| *Pparγ2* | GAGTAGCCTGGGCTGCTTTT | CTGATCACCAGCAGAGGTCC |
| *Cebp/α* | GACCATCCGCCTTGTGTGTA | CTGACATTGCACAAGGCACC |
| *Lpl* | TCGCCTGGTCGAAGTATTGG | TTGCATCCTGGCTGGAAAGT |

**Supplementary Table 1.** Sequence of Primers

| **Antibodies** | **Souse** | **Identifier** |
| --- | --- | --- |
| AR1 | Bioss | bs-0610R |
| AR2 | Proteintech | 14361-1-AP |
| GAPDH | Cell signaling | 5174 |
| ALP | Abcam | ab65834 |
| RUNX-2 | Abcam | ab23981 |
| OSX | Abcam | ab209484 |
| COL-1 | Abcam | ab34710 |
| OCN | Abcam | ab93876 |
| OPN | Abcam | ab8448 |
| TRAP | Abcam | ab235448 |
| CTSK | Abcam | ab19027 |
| NFATc1 | Cell signaling | 8032 |
| PPARγ2 | Santa Cruz Bio | sc-166731 |
| CEBP/α | Santa Cruz Bio | sc-166258 |
| LPL | Abcam | ab21356 |

**Supplementary Table 2.** Antibody list.
